# Applications of Isosceles Triangular Coupling Structure in Optical Switching and Sensing

**DOI:** 10.3390/s24248221

**Published:** 2024-12-23

**Authors:** Lili Zeng, Xingjiao Zhang, Qinghua Guo, Yang Fan, Yuanwen Deng, Zhengchao Ma, Boxun Li

**Affiliations:** 1 New Energy Institute, Hunan Vocational Institute of Technology, Xiangtan 411104, China; zenglili8368@126.com (L.Z.);; 2 School of Mechatronics Engineering, Ping Xiang University, Pingxiang 337055, China; 3 School of Physics and Optoelectronics, Xiangtan University, Xiangtan 411105, China

**Keywords:** liquid crystal, optical switching, sensing

## Abstract

In the case of waveguide-based devices, once they are fabricated, their optical properties are already determined and cannot be dynamically controlled, which limits their applications in practice. In this paper, an isosceles triangular-coupling structure which consists of an isosceles triangle coupled with a two-bus waveguide is proposed and researched numerically and theoretically. The coupled mode theory (CMT) is introduced to verify the correctness of the simulation results, which are based on the finite difference time domain (FDTD). Due to the existence of the side mode and angular mode, the transmission spectrum presents two high transmittance peaks and two low transmittance peaks. In addition, the four transmission peaks exhibit different variation trends when the dimensions of the isosceles triangle are changed. The liquid crystal (LC) materials comprise anisotropic uniaxial crystal and exhibit a remarkable birefringence effect under the action of the external field. When the isosceles triangle coupling structure is filled with LC, the refractive index of the liquid crystal can be changed by changing the applied voltage, thereby achieving the function of an optical switch. Within a certain range, a linear relationship between refractive index and applied voltage can be obtained. Moreover, the proposed structure can be applied to biochemical sensing to detect glucose concentrations, and the sensitivity reaches as high as 0.283 nm·L/g, which is significantly higher than other values reported in the literature. The triangular coupling structure has advantages such as simple structure and ease of manufacturing, making it an ideal choice for the design of high-performance integrated plasmonic devices.

## 1. Introduction

As is well known, surface plasmon polaritons (SPs), which propagate on the metal and dielectrical interface and can overcome the diffraction limit [1,2,3], have been widely used in biochemical sensing, information technology, and so on [4,5]. They demonstrate the perfect characteristics of breaking conventional diffraction limits and controlling the propagation of the light signal on the nanometer scale [6,7,8]. Owing to their excellent characteristics, SPs have aroused great interest among researchers, and SPs have been widely applied in plasma filters [9,10,11], optical switches [12,13,14], and sensors [15,16,17,18,19].

Metal–insulator–metal (MIM) waveguide structures have attracted attention and great interest from a wide range of researchers due to their advantages of simple preparation and deep sub-wavelength confinement [20]. Some applications based on MIM structures have been researched in detail, such as those based on filtration [21], sensing [22,23], hemoglobin detection [24], detection of cancer biomarkers [25], and so on. For example, Grineviciute et al. proposed and realized experimentally a thin dielectric film which results from Fano-like resonant coupling. And the results show that the film resulting from the Fano-like coupling has extremely strong sensitivity, especially to the incidence angle [21]. Chou et al. designed a plasmonic sensor consisting of a bus waveguide and a circular resonator, resulting in a high sensitivity and immersion intensity of 2800 nm/RIU and 86.97%, respectively [22]. Zhu et al. investigated an MIM structure which was composed of SiO_2_ branches coupled with a cavity; the structure can act as a sensor, with sensitivity and FOM up to 0.45 nm/°C and 156.25 [23], respectively. A D-shape cavity and a waveguide coupled with a barrier have been proposed, and such a design can be used in hemoglobin detection for three different human blood groups [24]. Tathfif et al. designed a MIM sensor which contained a concentric triple resonator, the FOM, FOM* and Q-factor of which can reach up to 91.02, 0.26 × 10^6^ and 99.75, respectively [25]. The dual transparency effect can be observed in the U-shaped structure, which can act as a sensor with sensitivity and figure of merit up to 1225 nn/RIU and 62.5, respectively [26]. It is worth noting, however, that once the MIM structure has been fabricated, it is not tunable, which hinders its further application. It is necessary to design a device the optical characteristics of which can be continuously tuned to extend its performance.

In this paper, we research an isosceles triangular coupling structure numerically and theoretically. The CMT demonstrates that the simulation results agree well with the calculated results. Due to the presence of side modes and angular modes, the transmission spectrum exhibits two high transmission peaks and two low transmission peaks. Furthermore, the trends of the four transmission peaks differ when the size of the isosceles triangle changes. When a liquid crystal fills an isosceles triangular coupling, the refractive index of the liquid crystal can be changed by changing the applied voltage, thereby achieving the function of an optical switch. The triangular coupling structure can be applied in biochemical sensing to detect glucose concentration, and its sensitivity is as high as 0.283 nm·L/g. The isosceles triangular coupling structure, with its advantages of simple and easy fabrication, provides an excellent candidate for the design of high-performance integrated plasmonic devices.

## 2. Structural Model and Analytical Method

**Figure 1:**
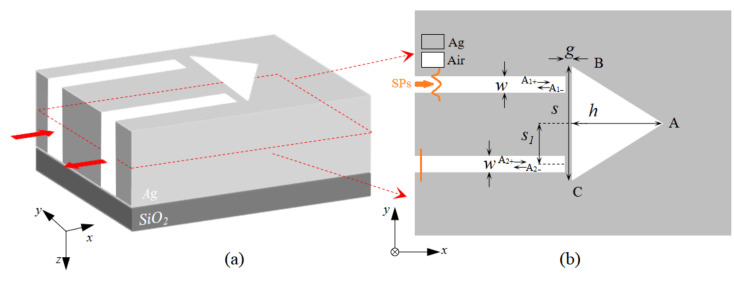
The diagram of the isosceles triangular coupling structure: (**a**) 3D structure, (**b**) 2D structure (top view).

Figure 1 shows the diagram of an isosceles triangular coupling structure, which consists of an isosceles triangle coupled with a two-bus waveguide, in which the width of the bus waveguide is represented by *w*, and the base and height of the isosceles triangle are denoted by *S* and *h*, respectively. The points of A, B and C stand for the three vertices of the isosceles triangle. Moreover, the distance between the isosceles triangle and the bus waveguide is *g*. *S*_1_ denotes the center distance between the bus waveguide and the isosceles triangle. The finite difference time domain (FDTD) is introduced to investigate the properties of the triangular coupling structure. The results are determined by using the software (ANSYS Lumerical FDTD 2024 R2.3). To optimize the computation, the absorbing boundary conditions of the perfectly matched layer (PML) and a periodic boundary along the *y*-direction are introduced. The white area is air and the gray area is silver; this can be expressed by the Drude model [27], and the equation can be written as *ε*(*ω*) = *ε_∞_* − *ω_p_*/(*ω^2^* + *iωγ_p_*), where *ω* stands for the angle frequency, *ε_∞_* = 3.7, *ω_p_* = 9.1*ev* denotes the bulk plasmon frequency and *γ_p_* = 0.018*ev* represents the damping rate. In order to reduce computational complexity, the silver layer is defined with sufficient thickness. The silver layer is then deposited on a SiO_2_ substrate. Unless otherwise specified, some parameters of the article species remain unchanged, such as that the width of the bus waveguide *w* is 50 nm, the distance g is 20 nm and the temperature is kept at 20 °C. During the calculation process, the mesh are set as Δ*x* = Δ*y* = 3 nm, and the temporal steps are set as Δ*t* = Δ*x*/2*c*. Using the existing micro–nano processing technology, the isosceles triangular coupling structure can be easily prepared. The preparation process of the structure is elaborated below. Firstly, the silver film is deposited on the silicon dioxide substrate by magnetron sputtering or e-beam evaporation; after that, the photoresist is applied to the surface of the silver layer. A mask plate with an isosceles triangular coupling structure is introduced, and the lithography process begins. The unwanted resin is removed by etching O_2_ using plasma etching. Furthermore, the exposed silver layer can be etched away by reactive ion etching. At the final step, the ashing process is used to remove the remaining photoresist, and the isosceles triangular coupling structure has been fabricated.

To research the properties of the isosceles triangular coupling structure, the coupled mode theory (CMT) is introduced to numerically study the optical characteristics of the structure. It is clear from Figure 1b that the input and output SPs in the bus waveguide are denoted by *A_i__±_*(*i* = 1 or 2), respectively. The + indicates that light is incident into the isosceles triangle, and − indicates that light is reflected away from the isosceles triangle. When the SPs with a frequency ω is transmitted from the input port, at this point, the output waveguide has no incident light, which means that *A_2+_* = 0; the amplitude of isosceles triangle *A* can be expressed as [16,28]

(1) $$\frac{dA}{dt}=(-j\omega_{1}-\frac{1}{\tau_{0}}-\frac{2}{\tau_{1}})A+\sqrt{\frac{2}{\tau_{1}}}A_{1+}$$

(2) $$A_{1-}=-A_{1+}+\sqrt{\frac{2}{\tau_{1}}}A$$

(3) $$A_{2-}=-A_{2+}+\sqrt{\frac{2}{\tau_{1}}}A$$

Here, *ω*_1_ represents the resonance frequency of the isosceles triangle. *τ*_0_ stands for the decay time of internal loss of the isosceles triangle, and we assume equal decay times for the coupling between the isosceles triangle and the two bus waveguides; this is denoted by *τ*_1_.

According to the above equations, it is concluded that the transmittance can be simplified as

(4) $$T={\left|\frac{A_{2-}}{A_{1+}}\right|}^{2}={\left|\frac{\frac{2}{\tau_{1}}}{-j(\omega -\omega_{1})+\frac{1}{\tau_{0}}+\frac{2}{\tau_{1}}}\right|}^{2}$$

From the above expression of transmittance, it can be seen that when the frequency of the incident light matches the intrinsic frequency of the isosceles triangle, the corresponding transmittance reaches the maximum. At this time, the value of the transmittance mainly depends on *τ*_0_ and *τ*_1_. The size of the isosceles triangle has been determined, which means that *τ*_0_ has been determined, and *τ*_1_ is mainly determined by the distance between the waveguide and the isosceles triangle. The transmittance can be adjusted by changing this spacing.

## 3. Results and Discussion

**Figure 2:**
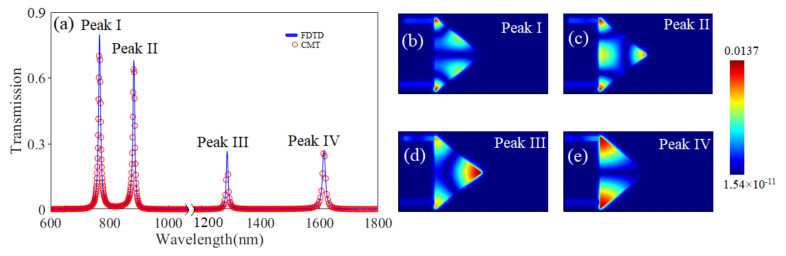
(**a**) The transmission spectrum of the isosceles triangular coupling structure. (**b**–**e**) are the distributions of magnetic field with four peaks. The coordinates of the three vertices of the isosceles triangle are as follows: A (100,0), B (−500,480) and C (−500,−480).

Figure 2a shows the transmission spectrum of the isosceles triangular coupling structure. It is apparent that there are four peaks, which are located at the wavelengths of 768 nm, 885 nm, 1290 nm and 1617 nm, respectively, and labeled as Peaks I, II, III and IV. It is clear that the transmittance of Peaks I and II are higher than those of Peaks III and IV. In addition, the CMT results marked with red circles agree very well with the simulation results marked with a solid blue line. To explore the mechanisms of the transmission properties, the distributions of magnetic field within the four peaks have been drawn in Figure 2b–e. It can clearly be observed from Figure 2b that most of the energy is concentrated in the two base angles and the two hypotenuses, which are defined as the angular and side modes, respectively. There are three angular modes and one side mode in Figure 2c, where the energy is mainly distributed in three corners together with the bottom edge. Similarly to Figure 2c, most of the energy is distributed in the three corners in Figure 2d; just to be clear, the energy in angle A is much higher than the energy in angles B and C. As to Peak IV, the energy is mainly distributed in the two corners of the bottom edge, and two angular modes can be observed.

**Figure 3:**
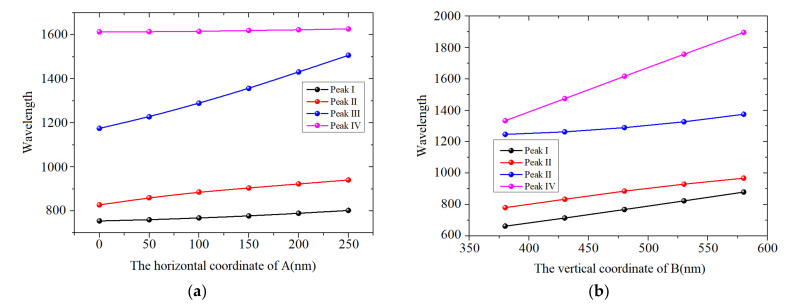
The coordinates of the three vertices of the isosceles triangle are as follows: A (100,0), B (−500,480) and C (−500,−480). The effects of structural parameters on transmission characteristics are shown here: (**a**,**b**) are the horizontal coordinates of A and B/C.

Based on the distributions of the magnetic fields in the four peaks, it is concluded that there is a great influence on the distribution of the energy field, relative to the structure’s parameters. And the effects of the base and height dimensions of the isosceles triangle on the transmission characteristics is studied in detail. Figure 3 shows the wavelength variations of the four peaks, with different coordinates of A and B/C. It is observed from Figure 3a that the wavelength shifts of Peaks I and IV are much smaller than those of Peak II and III. The results are consistent with the distributions of magnetic field shown in Figure 2b–e. It is clear from Figure 2c,d that most of the energy is concentrated in the three corners, while there is no energy distribution in angle A in Figure 2b,e. The result means that the change of isosceles triangle height has a greater influence on Peaks II and III than on Peaks I and IV. Figure 3b shows that the four peaks will move toward longer wavelengths, as the vertical coordinate of B increases from 380 nm to 580 nm. In addition, the wavelength shift of Peak IV is the largest, relative to Peaks I, II and III. According to the four magnetic field distributions in Figure 2b–e, compared to other figures, the energy distributions in Figure 2e are strongest in the two base corners; this shows that the change of the bottom edge has the greatest influence on Peak IV. The analysis of this finding is consistent with the above analysis.

**Figure 4:**
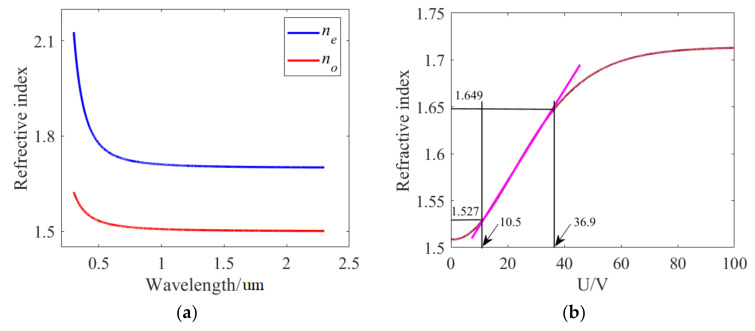
(**a**) Extraordinary and ordinary refractive indices, *n_e_* and *n_o_*, with various wavelengths. (**b**) The refractive index of LC with different voltages.

The liquid crystal (LC) materials comprise an anisotropic uniaxial crystal and exhibit a remarkable birefringence effect under the action of external field; they are widely used in various optical devices. This is due to the rearrangement of liquid crystal molecules under the action of electric or magnetic fields, which changes their optical properties. The refractive indices can be affected by two important factors: the wavelength and temperature. The extraordinary and ordinary refractive indices, which are denoted as *n_e_* and *n_o_*, respectively, can be expressed as follows [29]:

(5) $$n_{e}=A_{e}+B_{e}/\lambda^{2}+C_{e}/\lambda^{4}$$

(6) $$n_{o}=A_{o}+B_{o}/\lambda^{2}+C_{e}/\lambda^{4}$$

where the coefficients *A_e_*, *B_e_*, *C_e_* and *A_o_*, *B_o_*, *C_o_*, which can be obtained in Ref. [30], are 1.6993, 0.0085 and 0.0027 and 1.4998, 0.0067 and 0.0004, respectively. Figure 4a shows the extraordinary and ordinary refractive indices with different wavelengths. Within the short wavelength, the two refractive indices *n_e_* and *n_o_* decrease sharply with the increase of the wavelength, and with the further increase of the wavelength, the two refractive indices *n_e_* and *n_o_* decrease slightly and tend to flatten, with values of 1.72 and 1.51, respectively. Ultimately, the refractive index of LC can be described as the following [31]:

(7) $$n=\frac{n_{o}n_{e}}{\sqrt{n_{e}^{2}{cos}^{2}\theta +n_{o}^{2}{sin}^{2}\theta }}$$

Here, *θ* stands for the included angle between the long axis of the LC molecule and the x-axis, which can be described by the following equation [32]:

(8) $$\theta =\left\{\begin{array}{l} 0\;\;\;\;\;\;\;\;\;\;\;\;\;\;\;\;\;\;\;\;\;\;\;\;\;\;\;\;\;\;\;\;\;\;\;U\leq V_{th}\\ 0.5\pi -2\tan^{-1}\left\{\exp[-\right.\left.\left(\frac{U-V_{th}}{30V_{th}}\right)\right\}\;\;\;\;\;\;U\geq V_{th}\; \end{array}\right.$$

**Figure 5:**
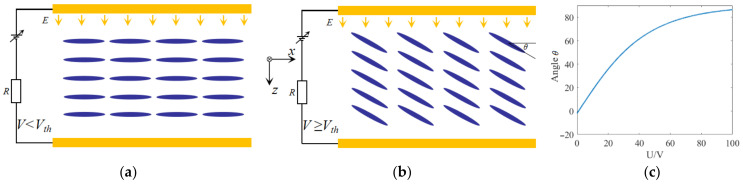
The configurations of LC molecules with different voltages: (**a**) *V* < *V_th_*, (**b**) *V* ≥ *V_th_*. (**c**) The angle *θ* between the long axis of the LC molecule and the x-axis, with different voltages.

The included angle *θ* can be seen from Figure 5b. Here *V_th_* represents the threshold which can be obtained from the following equation [33]:

(9) $$V_{th}=\pi \sqrt{\frac{k_{11}}{\Delta \varepsilon \varepsilon_{0}}}$$

Here, *k_11_* denotes the splay elastic constant of LC, ∆*ε* and *ε_0_*, respectively, stand for the electric constant and anisotropy dielectric permittivity, *ε_0_* = 8.85pF/m and ∆*ε* = *ε_ǁ_ − ε*_⊥_; the values for *k_11_*, *ε_ǁ_* and *ε*_⊥_ can be obtained in Ref. [34]. It is concluded that the value of *V_th_* is approximately 0.95 V. It is found from Equation (8) that when the applied voltage is less than the threshold voltage, the arrangement of the LC molecules is not affected, and the included angle θ remains basically unchanged; its value is kept at 0, as shown in Figure 5a. While *V* ≥ *V_th_*, the angle *θ* will increase with the increase of applied voltage, which can be seen from Figure 5b,c. Finally, the angle *θ* will infinitely close to 0.5π, which means that the long axis of the LC molecule will be parallel to the direction of the electric field; at this time, the refractive index of the LC reaches its maximum value *n_e_*. In this study, we assume that the long axis of the LC molecule is originally along the *x*-axis (*θ* = 0); to ensure this, a lens cleaning tissue is introduced to brush the PVA layer hundreds of times along the *x*-direction [35,36].

**Figure 6:**
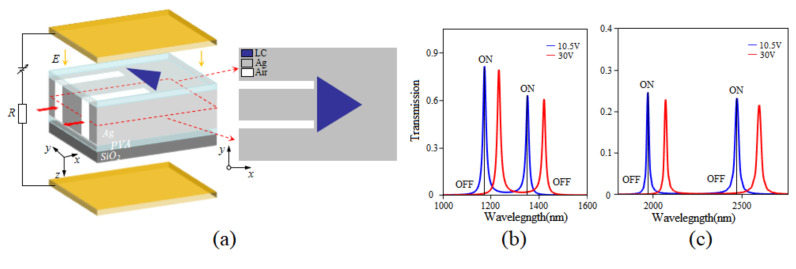
(**a**) The diagram of isosceles triangular coupling structure filled with LC. (**b**,**c**) The transmission spectra with different voltages applied.

When the isosceles triangular coupling structure is filled with LC, an external voltage with an electric field direction along the z−axis can be applied to adjust the refractive index of the LC, as shown in Figure 6a. At the beginning, owing to the fact that a lens cleaning tissue owing to the fact that a lens cleaning tissue brush the polyvinyl alcohol (PVA) layer repeatedly, the angle *θ* = 0, it is obtained from Equation (7) that the refractive index of LC is equal to *n_o_*. When the applied voltage exceeds the threshold voltage and continues to increase, the angle *θ* increases to 90 degrees, at which point the refractive index of the LC becomes as high as *n_e_*. In other words, it is feasible to adjust the refractive index by altering the angle through changing the applied voltage. As demonstrated in Figure 4b, there is a linear relationship between the refractive index and the applied voltage when the refractive index is varied from *n_o_* to *n_e_*, as expressed in the equation *n* = 0.0045*U* + 1.483. Within a given wavelength range, it is possible to achieve the “ON” and “OFF” states of any wavelength, thereby realizing the function of an optical switch. Figure 6b,c show the transmission spectra at applied voltage levels of 10.5 V and 30 V, respectively. We set a threshold that corresponds to state “ON” when the transmittance is higher than 10% and one corresponding to state “OFF” when the transmittance is lower than 10%. When the applied voltage is 10.5V, the refractive index is approximately 1.53, and Peaks I, II, III and IV show transmittance levels of 87%, 63%, 26% and 25%, which can be defined as state “ON”. When the voltage increases to 30 V, the refractive index is approximately 1.633, and the four peaks shift towards longer wavelengths. The transmittance of the original four peaks becomes very low, corresponding to the state “OFF”. The results indicate that this structure can serve as an optical switch capable of achieving an ON/OFF state at any wavelength by adjusting the applied voltage.

SPs are well known to be very sensitive to changes in the ambient medium and their excellent properties can be used in sensing applications [37,38,39,40]. In the field of biochemistry, these sensors can be used to measure the concentrations of solutions such as glucose, sodium chloride and sucrose. Glucose in the body is the main source of energy for the cells. The balance of glucose in the body is very important for maintaining normal physiological functions, and any imbalance may trigger a range of health problems. Therefore, it is important to understand and manage blood glucose levels, especially for those with diabetes or other related conditions. Water is an important part of the human body, so studying the concentrations of different substances in an aqueous solution is of great significance in biomedicine. The refractive index of glucose solution is closely related to its concentration, which can be expressed as [40]

(10) n=0.00011889C+1.33230545

**Figure 7:**
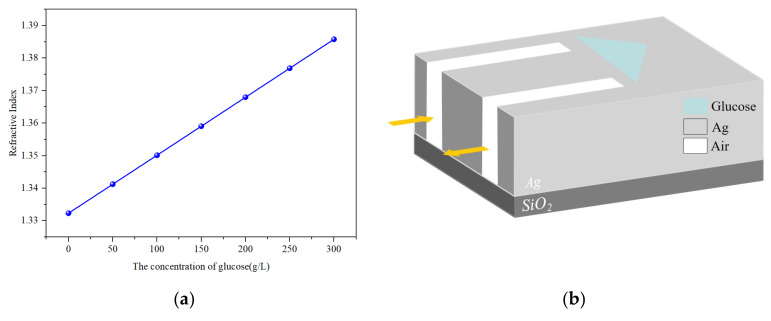
(**a**) The refractive index as a function of the concentration of glucose solution. (**b**) The glucose concentration detection model.

Here, *C* represents the concentration of the glucose solution. It can be clearly seen from Figure 7a that there is a linear relationship between the refractive index and the concentration of the glucose solution. As the concentration of glucose increases from 0 to 300 g/L, the corresponding refractive index increases from 1.3323 to 1.3858. When the isosceles triangle is filled with glucose solution, as shown in Figure 7b, we can determine the concentration of the glucose solution by the wavelength shift.

**Figure 8:**
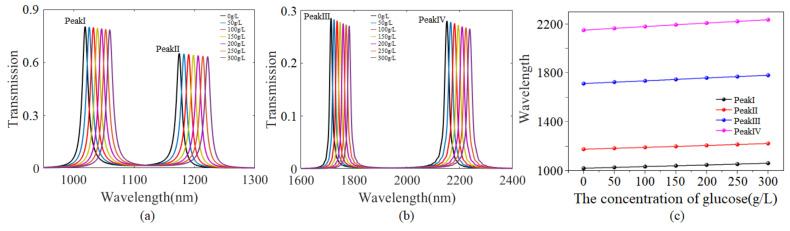
(**a**,**b**) The transmission spectra associated with glucose solutions of different concentrations. (**c**) The wavelength shifts with glucose solutions of different concentrations.

Figure 8a,b exhibit transmission spectra for glucose solutions of different concentrations. As the concentration of the glucose solution increases from 0 to 150 g/L, and then to 300 g/L, the corresponding refractive index increases from 1.3323 to 1.3591, and then to 1.3858, and the resonant wavelength of Peak I moves from 1018.8 nm to 1039.3 nm, and then continues to increase to 1059.7 nm. Similarly, as the concentration of glucose solution increases, the resonant wavelengths of Peaks II, III, and IV move toward longer wavelengths. It should be pointed out that as the resonant wavelengths of four peaks move to longer wavelengths, the corresponding transmittance gradually decreases, but the decrease is not large. It is clear from Figure 8c that a linear relationship can be obtained between the wavelength shift and the concentration of the glucose solution. The sensitivity is defined as *S* = Δ*λ*/Δ*C*, according to the slope, and the sensitivities of Peaks I, II, III and IV are 0.136 nm·L/g, 0.156 nm·L/g, 0.228 nm·L/g and 0.283 nm·L/g, respectively. Obviously, it is found that Peak IV is the most sensitive to changes in the concentration of the glucose solution. Table 1 displays the sensitivity-based comparison of this work with other published papers. In Ref. [40], a plasmonics sensor with high sensitivity and using N-Doped Silicon had been proposed and investigated in detail, and the maximum *S* was 0.21 nm·L/g, which is higher than reported in other sources in the literature. Apparently, the sensitivity of the isosceles triangular coupling structure is higher than those reported in previous literature. The isosceles triangular coupling structure has advantages such as simple structure, easy fabrication and high sensitivity, making it applicable in fields such as biochemical sensing, optical switching and integrated photonic circuits.

## 4. Conclusions

In summary, this paper proposes an isosceles triangular coupling structure composed of an isosceles triangle coupled with two bus waveguides and conducts both theoretical and numerical studies on it. The coupled mode theory verifys the calculation results, which are based on the FDTD. It is found that the structural parameters have significant impacts on the angular and side modes, resulting in four transmission peaks with different transmittances. By adjusting the refractive index through the application of an external voltage, the triangular coupling structure can be applied to optical switching, based on the properties of LC birefringence. Additionally, this structure can be used to detect glucose concentrations, with a sensitivity as high as 0.283 nm·L/g. The proposed structure, with high sensitivity and compactness, provides guidance for the design of integrated photonic devices and biochemical sensors.

## Figures and Tables

**Table 1:** The comparison of the sensitivity determined in this work with other published papers.

Structure	Materials	Year	*S*	Reference
Double half-ring resonator	Ag	2021	0.145 nm·L/g	[37]
MIM cavity	Ag	2021	0.19 nm·L/g	[38]
MIM waveguide with r-shaped resonator	Ag	2022	0.16 nm·L/g	[39]
Nano-ring resonator	N-doped Si	2024	0.21 nm·L/g	[40]
Isosceles triangular coupling structure	Ag	2024	0.283 nm·L/g	This work

## Data Availability

Relevant data are available upon reasonable request to the corresponding author.
